# Long COVID a New Derivative in the Chaos of SARS-CoV-2 Infection: The Emergent Pandemic?

**DOI:** 10.3390/jcm10245799

**Published:** 2021-12-11

**Authors:** Diego Fernández-Lázaro, Nerea Sánchez-Serrano, Juan Mielgo-Ayuso, Juan Luis García-Hernández, Jerónimo J. González-Bernal, Jesús Seco-Calvo

**Affiliations:** 1 Department of Cellular Biology, Histology and Pharmacology, Faculty of Health Sciences, University of Valladolid, Campus of Soria, 42003 Soria, Spain; 2 Neurobiology Research Group, Faculty of Medicine, University of Valladolid, 47005 Valladolid, Spain; 3 Microbiology Unit of the Santa Bárbara Hospital, Castille and Leon Health (SACyL), 42003 Soria, Spain; nsanchezser@saludcastillayleon.es; 4 Department of Health Sciences, Faculty of Health Sciences, University of Burgos, 09001 Burgos, Spain; jfmielgo@ubu.es (J.M.-A.); jejavier@ubu.es (J.J.G.-B.); 5 Molecular Mechanisms of Cancer Program, Institute of Molecular and Cellular Biology of Cancer, Spanish National Research Council (CSIC), University of Salamanca, 37007 Salamanca, Spain; jlgarcia@usal.es; 6 Department of Hematology, Institute of Biomedical Research of Salamanca (IBSAL), University Hospital of Salamanca, 37007 Salamanca, Spain; 7 Physiotherapy Department, Institute of Biomedicine (IBIOMED), University of Leon, Campus de Vegazana, 24071 Leon, Spain; jasecc@unileon.es; 8 Department of Physiology, Basque Country University, 48930 Leioa, Spain

**Keywords:** SARS-CoV-2, long COVID, symptomatology, therapy, pathways, biomarkers

## Abstract

Coronavirus disease 2019 (COVID-19) is a multisystem illness caused by Severe Acute Respiratory Syndrome Coronavirus 2 (SARS-CoV-2), which can manifest with a multitude of symptoms in the setting of end-organ damage, though it is predominantly respiratory. However, various symptoms may remain after acute SARS-CoV-2 infection, and this condition is referred to as “Long COVID” (LC). Patients with LC may develop multi-organ symptom complex that remains 4–12 weeks after the acute phase of illness, with symptoms intermittently persisting over time. The main symptoms are fatigue, post-exertional malaise, cognitive dysfunction, and limitation of functional capacity. Pediatric patients developed the main symptoms of LC like those described in adults, although there may be variable presentations of LC in children. The underlying mechanisms of LC are not clearly known, although they may involve pathophysiological changes generated by virus persistence, immunological alterations secondary to virus–host interaction, tissue damage of inflammatory origin and hyperactivation of coagulation. Risk factors for developing LC would be female sex, more than five early symptoms, early dyspnea, previous psychiatric disorders, and alterations in immunological, inflammatory and coagulation parameters. There is currently no specific treatment for LC, but it could include pharmacological treatments to treat symptoms, supplements to restore nutritional, metabolic, and gut flora balance, and functional treatments for the most disabling symptoms. In summary, this study aims to show the scientific community the current knowledge of LC.

## 1. Introduction

Since the end of 2019, Coronavirus disease 2019 (COVID-19), a multisystem illness caused by Severe Acute Respiratory Syndrome Coronavirus 2 (SARS-CoV-2), which can manifest with a multitude of symptoms in the setting of end-organ damage, though predominantly respiratory, has emerged [1]. SARS-CoV-2, with devastating consequences for humanity, has transformed normalcy for the world [2]. At least 200 million cases of COVID-19 disease and 4 million deaths have been reported [3]. To date, the current availability of drugs to treat SARS-CoV-2, such as the use of convalescent plasma, antiviral drugs, dexamethasone, monoclonal antibodies, and immunomodulators, could contribute to the control of SARS-CoV-2 infection, although their effectiveness is limited [4]. The majority of confirmed cases of COVID-19 by detection of viral RNA by Real Time Reverse Transcriptase-Polymerase Chain Reaction (RT-PCR) techniques [5] present with flu-like symptomatology, muscle aches, runny nose, sore throat, gastrointestinal symptoms, and loss of the senses of smell and taste [4]. However, 20% of patients develop severe symptoms associated with respiratory difficulties and pneumonia. In addition, coagulation disorders, septic shock, multiorgan failure, and complications secondary to a systemic inflammatory response are associated with increased mortality [6]. Recent evidence has demonstrated that a variety of symptoms may remain after acute SARS-CoV-2 infection and this condition is referred to as “Long COVID” (LC) [7].

In August 2020, post-acute symptoms of COVID-19 were already reported in patients being seen in primary care [8]. In September 2020, the World Health Organization (WHO) published an update recognizing the existence of long-lasting effects following SARS-CoV-2 infection [9]. It is from this document that the WHO urges governments to recognize the long-term effects of COVID-19 and to ensure access to health services (primary care, special care and rehabilitation) for these patients. As early as November 2020, the Centers for Disease Control and Prevention (CDC) (Atlanta, GA, USA) began reporting the full range of short- and long-term health effects associated with COVID-19 that have been updated. Subsequently, the National Institutes of Health (NIH) (Bethesda, Rockville, MD, USA) described the condition of LC as sequelae extending beyond four weeks after initial infection [10]. In addition, The National Institute for Health and Care Excellence (NICE) created in December 2020 a guideline for the management of the long-term effects of COVID-19 [11]. The Ministry of Health of the Government of Spain generated the document “Scientific and technical information Coronavirus disease, COVID-19” in January 2021, which offers a specific LC section explaining the following: “patients with LC exhibit compromise and deterioration in the structure and function of multiple organs, and that it affects many people causing great health and social impact in the pandemic”. This document makes a clear distinction between sequelae arising from severe acute illness and LC [12]. Already in February 2021, a new policy report from the European Observatory on Health Systems and Policies documented the responses to LC in different countries of the European Union (EU). In addition, this document discusses how patients, and healthcare professionals, are driving some of these responses. Finally, this paper includes a call for recognition of the wide range of health impacts of LC from people reporting ongoing symptoms at 12 weeks post-SARS-CoV-2 infection [13]. Therefore, there is a need for information on LC for all patients (who have seen their quality of life completely diminished after infection and who for a long time have not been recognized as sick) and health professionals to try to reduce the uncertainty of action and help make decisions in the clinical setting.

## 2. Material and Methods

### 2.1. Search Strategy

This study is a narrative review that sought to evaluate updated information on a new and emerging condition, which has been described with various terms “Persistent COVID”, “Long COVID” or “Chronic COVID”. A search was carried out between June and September 2021 in the databases: Medline (PubMed), SciELO, and Cochrane Library Plus. Plus. Several terms (Mesh) were used as keywords for the search: Long COVID, Persistent COVID, COVID long-effects, COVID long-term effects, post COVID, COVID complications, COVID recurrent, COVID sequelae, and chronic COVID. The Boolean operators “AND” and “OR” were used as a search nexus. The search was completed with documents from official Spanish and international institutions.

After searching the articles in the databases, the search titles were cross-checked to identify duplicates and potential publications to add. After reading the abstract, a full text review of the selected articles was performed.

### 2.2. Inclusion and Exclusion Criteria

The following inclusion criteria were applied to select articles: (i) Full text access; (ii) being a review, clinical trial, observational study, case report/study; (iii) articles that identified or provided information about: pathways, etiology, symptomatology, biomarkers, disability, and pediatric at a later stage than acute COVID-19; (iv) human studies; (v) papers whose publication date was between 2020 and 2021; (vi) languages were restricted to English, German, French, Italian, Spanish, and Portuguese. Regarding exclusion criteria, the criteria applied were: (i) Publications not related to Long COVID; (ii) duplicate papers; (iii) animals’ studies; (iv) articles published before 2020.

## 3. Description of Long COVID (LC)

**Figure 1:**
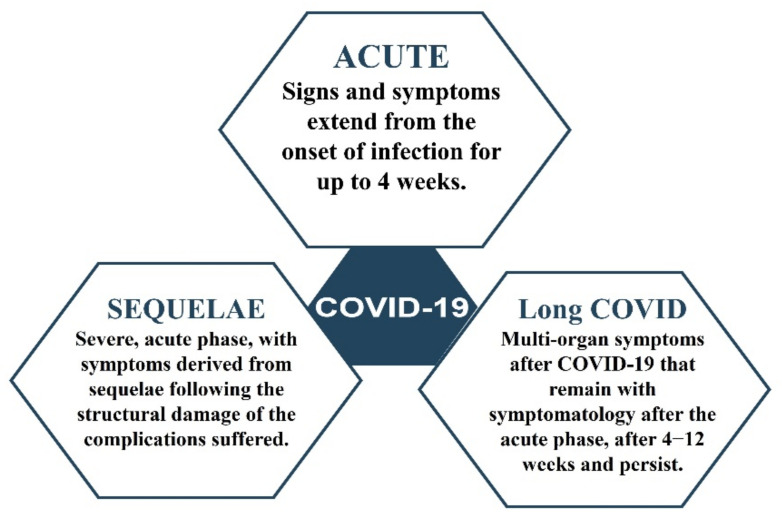
Long COVID, Sequelae of COVID-19 and Acute COVID-19.

It is necessary to have clear concepts for the diagnosis, identification, treatment, management, and handling of LC patients to distinguish it from other pathologies related to SARS-CoV-2 infection. To date, it is possible to differentiate between acute COVID-19, sequelae derived from COVID-19 and LC (Figure 1). The patient with acute COVID-19 is one who manifests signs and symptoms of COVID-19 from the onset of infection, and which may extend up to 4 weeks after infection. The patient with sequelae of COVID-19, often referred to as post-COVID, may have a history of severe acute involvement by SARS-CoV-2 infection. Given the severity of the infection, these patients may have increased risk factors for hospital admission and that they present symptoms derived from sequelae following the structural damage of the complications suffered. The general features presented by this type of patient with sequelae are adult males (65–75 years) with associated comorbidities. All patients with sequelae have had access to RT-PCR for the diagnosis of the infection, when they received hospital care, and have received advanced health care and remained under follow-up in hospital consultations [14].

On the other hand, the patients with LC may develop multi-organ symptom complex that remains 4–12 weeks after the acute phase of illness, with symptoms intermittently persisting over time and cases are not considered to have a disease-free period, although the clinical picture is fluctuating [15]. From the clinical point of view, the symptomatology does not disappear; it may be changeable or there may be outbreaks, but there is no clinical endpoint. Therefore, there is no period of healing of the acute phase and there is no post-COVID moment. Most LC patients were infected in the first months of 2020, i.e., in the first wave of the pandemic, and their diagnosis by RT-PCR during the acute phase of COVID-19 was only confirmed when it became accessible. Moreover, in LC patients with a limited medical evaluation access (e.g., telehealth), their COVID-19 diagnosis was subsequently confirmed by validated cellular immunity laboratory studies [16]. The patients with LC are the “great forgotten” of the health system and there is no explanation for an alternative underlying disease. It would be necessary to study whether in the patient with LC there is any biomarker or alteration in tissues or organs that is not detected by routine diagnostic tests. This would entail performing other more specific tests [17].

There may be an overlap in the interpretation between acute LC and post-COVID-19. With respect to acute post-COVID-19, there is currently no evidence for specific physiological changes (predictive of chronicity) at 12 weeks, and therefore it would be preferable to use the term persistent COVID-19 for symptoms of any duration beyond 4 weeks. In addition, the use of the prefix “post” implies that the acute infection and any active disease process have resolved, which is currently unknown. Thus, in acute post-COVID-19, there is a nuance that differentiates it from LC, and at a time when the clinical COVID has disappeared, patients are truly post-COVID. However, in the case of patients with LC, it is not possible to identify, beyond the fluctuation of symptoms, a moment in which the disease is overcome, i.e., a post-COVID moment. In addition, post-acute manifestations present as residual symptoms that persist after recovery from acute infection; organ dysfunction that persists after initial recovery; and new syndromes that develop after an initially asymptomatic or mild infection. The patient with acute post-COVID-19 may present with additional symptoms that persist over time in addition to their sequelae. However, the clinical presentation of the LC patient is not compatible with post-COVID-19 because, although there is no fixed pattern in all patients, the symptomatology often presents with flare-ups in which symptoms may become more severe and new clinical manifestations may appear [14,15,16].

The global standardization of population health diagnostic information carried out by the WHO, through the International Classification of Diseases (ICD) in its eleventh edition, which comes into force in 2022, includes the following codes in reference to the identification of LC: (a) RA02: Post-COVID-19 disease; and (b) RA03: Multisystem inflammatory syndrome associated with COVID-19 [18]. This identification is probably insufficient, and it is necessary to establish a code that specifically denominates them and differentiates them from other entities that can occur in post-COVID-19 disease or from other associated multisystemic inflammatory syndromes. In this sense, we could follow those guides established by the National Institute for Health and Clinical Excellence, Scottish Intercollegiate Guidelines Network and Royal College of General who consider two entities within the definition of prolonged COVID-19 [11]: continuous symptomatic COVID-19: signs and symptoms of COVID-19 from 4 to 12 weeks; Post COVID-19 syndrome: signs and symptoms that develop during or after a COVID-19 compatible infection, continue for more than 12 weeks and are not explained by an alternative diagnosis. In addition, the French Haute Autorité de Santé specifies three criteria to identify cases suffering from “Symptômes Prolongés COVID-19” [19]: having presented with a symptomatic form of COVID-19, continuing with one or more of the initial symptoms 4 weeks later, and these symptoms that cannot be explained by any other diagnosis.

## 4. Mechanism of Action of SARS-CoV-2 in the Etiopathogenesis of Long COVID (LC)

The underlying mechanisms in LC are not clearly known, although some hypotheses have been established that could involve the following: pathophysiological changes generated by the persistence of the virus located in tissue reservoirs or cells of the immune system where it would remain active, immunological alterations secondary to the virus–host interaction that would generate an aberrant immune response, tissue damage of inflammatory origin that continues after the response to acute infection and by the hyperactivation of coagulation and platelets, although each clinical symptom of LC could have its own pathways [7,20].

### 4.1. Alterations of the Immune Response

#### 4.1.1. Dysregulation of the Immune System

Altered immune response in LC patients due to persistence of infection results in inadequate innate immune response, reduced activity of the interferon system, pathological changes in inflammatory mechanisms, malfunction of macrophages in early stages, alterations in the induction of adaptive immune response through stimulation of effector T cells with proinflammatory properties and ineffective in eliminating SARS-CoV-2 [14,20,21].

Dysregulation of the immune system in LC patients is characterized by an increase in Interferon gamma (IFN-γ) and Interleukin (IL)-2 cytokines, pathological changes in populations of CD4+, CD8+ lymphocyte subpopulations, the monocytic CD14+ and CD16+ subset, deficits of B lymphocytes and monocytes, alterations in the cellular response to SARS-CoV-2 antigens (S, M, N, P proteins) and decreased levels of the Chemokine (C-C motif) ligands 4 (CCL4) [21]. All this presents different profiles of clinical relevance, with an inflammatory profile marked by a decrease in CD4+ and an increase in IFN-γ, CD8+ and Natural Killer (NK) cells. On the other hand, the immune profile is characterized by an increase in CD4+ and CD8+ cells [22]. The immune response in LC patients induces the activation of effector T cells with proinflammatory properties and the ability to generate an effective immune response to eliminate the virus, but without adequate recruitment signals to attract activated T cells [21]. Recovered COVID-19 patients had elevated levels of proinflammatory IL-17A, stem cell factor (SCF), IL-12p70, IL-1β, macrophage inflammatory protein-1 (MIP-1β), and pro-angiogenic macrophage inflammatory protein 1β, brain-derived neurotrophic factor, and vascular endothelial growth factor at 6 months after infection compared to subjects without disease [23]. This could reflect that in recovered patients there is a pattern of chronic inflammation and angiogenesis. These alterations of the immune system are different from COVID-19 sequelae patients where excessive inflammation predominates with plasma levels of IL-6 and IL-10, and an increased immune activity rate for activation, recruitment, and modulation processes which take place to restore dysregulated T cells [14].

The persistence of SARS-CoV-2 infection could be caused by an inadequate or deficient innate immune response in the interferon production and secretion system, pathologically increased inflammatory mechanisms, and dysfunction of macrophage activity in the early stages of infection. In addition, the alteration of the adaptive immune response, manifested by a marked lymphopenia with disproportion in the concentrations of lymphocyte subpopulations in favor of virgin cells that follow inadequate temporal kinetics, would explain the unfavorable evolution of some patients [7,21]. Moreover, the cause of the inadequate immune response in LC patients could be a consequence of alterations in the genetic component of immunity—haplotype of the major histocompatibility complex (MHC) or of the existence of polymorphisms with phenotypic alterations in the genes that give rise to the appearance of a pathological immune response [24]. Immunologic recovery after COVID-19 is complex, with profound persistent cellular abnormalities that correlate with a change in the nature of the inflammatory response. Increased oxidative phosphorylation and reactive oxygen species-associated inflammation replace those driven by TNF-α and IL-6, establishing changes in late immune-metabolic inflammatory patterns, which in conjunction with unresolved immune cell defects, if maintained, would contribute to persistent symptoms and development of LC [25].

#### 4.1.2. Presence of Autoantibodies

The participation of B cells in autoimmunity must be considered in the etiopathogenesis of LC. In this sense, the presence and high reactivity of autoantibodies in SARS-CoV-2 infection has been described that would act against immunomodulatory proteins (cytokines/chemokines; complement components and cell surface proteins) and would result in the perturbation of immune function [22,26]. In fact, autoantibodies against interferons, neutrophils, connective tissues, cyclic citrullinated peptides and cell nuclei have been identified in serological samples in 10–50% of moderate and severe patients with COVID-19 [26] and elevated concentrations of antiphospholipid autoantibodies in 52% of severe patients hospitalized with SARS-CoV-2 infection [27]. Therefore, the presence of these autoantibodies would condition the inadequate immune response and impair virological control by inhibiting immunoreceptor signaling and altering the composition of peripheral immune cells. All this contributes decisively to the immunopathology of COVID-19, aggravating its symptoms or maintaining them over time [28]. In this sense, other diseases of autoimmune origin such as lupus and rheumatoid arthritis develop symptoms (fatigue, joint pain, concentration difficulties and headache), which are very similar to those suffered by LC patients [22].

#### 4.1.3. Thyroid Dysfunction

Altered thyroid function has been reported in 15–20% of patients with COVID-19. Thyroid dysfunction could play a role in the etiopathogenesis of LC-associated autoimmunity [29,30]. Altered thyroid activity would cause autoreactive T cells to escape negative selection in the thymus, i.e., SARS-CoV-2 would act by stimulating the loss of the peripheral tolerance system in the thyroid. Thus, T cells would contribute to tissue injury and would be responsible for the pathophysiology of LC in a similar manner in autoimmune diseases [29].

#### 4.1.4. Nutritional Deficiencies

Specialized pro-resolving mediators (SPMs) are produced by cells of the innate immune system [31]. SPMs are synthesized from the stereoselective enzymatic conversion of essential fatty acids (arachidonic acid, eicosapentaenoic acid (EPA), docosapentaenoic acid ϖ-3, and docosahexaenoic acid (DHA)) [32]. For EPA and DHA, anti-inflammatory properties have been proposed because they compete with arachidonic acid in reducing proinflammatory eicosanoids [31]. SPMs are grouped into four families: lipoxins, resolvins, proteins and maresins. These endogenous mediators share basic physiological properties in regulating host responses to actively enhance the resolution of infectious inflammatory response mechanisms, such as the following: reducing host proinflammatory cytokine/chemokine production, limiting neutrophil trafficking, stimulating phagocytosis of apoptotic cells by macrophages, killing bacteria, and degrading cellular debris through G protein-coupled receptors (GPCRs), and protecting organs and/or tissues [31,32].

In COVID-19, SPMs could stimulate the resolution of lung inflammation and reduce tissue damage in patients by early termination of SARS-CoV-2 infection to stop the cytokine storm. In addition, it could be used in decreasing chronic inflammation in the post-acute phase, which would generate a potential use of SPMs in LC [33,34]. In LC patients, nutritional profiles are unbalanced, favoring proinflammatory lipid mediators over SPMs [33]. In other words, nutritional strategies could be considered to increase endogenous production of SPMs or their supply in sufficient quantities to allow them to develop their function [33].

On the other hand, a high percentage of COVID-19 patients hospitalized during the acute phase were deficient in vitamin D and omega-3 fatty acids [35,36]. Thus, it could be hypothesized that it would be advisable to follow a healthy diet rich in food that provides omega-3, vitamin C, vitamin D, Zinc and Selenium to attenuate the symptoms of LC, since all the targets for the origin of this syndrome point to the immune system [37]. Regarding vitamin D, there are currently two theories regarding the recommendation to increase its intake (mainly with supplements) to prevent or treat LC: (i) there is insufficient evidence to adopt a nutritional recommendation; (ii) recommend vitamin D supplementation above the recommended daily intakes because high levels of vitamin D have been correlated with lower rates of infection and a lower risk of hospitalization, due to the direct relationship with immunity and its effects of modulating tissue damage, mainly at the muscular level [38]. As for omega-3 fatty acids, the most recent evidence has demonstrated the important role they play in the resolution of inflammation of infectious etiology, as a substrate of the MPE, which is implicated in many of the symptoms of la LC [33,34]. The use of therapeutic nutrients to improve and/or reinforce the diet in the recovery phase of the disease is also recommended, as well as the intake of vitamin complexes [37]. In this regard, supplementation with B-complex vitamins, specifically vitamin B_12_, is recommended, since it has been shown that people with LC had low reserves of this vitamin before becoming infected with SARS-CoV-2, which could favor the chronification of symptoms [26]. The administration of the glycophosphopeptide food supplement AM3 (an immunomodulatory agent) has been proposed to attenuate the inflammatory response, modulate the immune response and attenuate muscle damage in SARS-CoV-2 infection [4]. Pending further research that will shed lighter and specify the benefits that certain nutrients may have in the prevention of LC and the improvement of its symptoms, the best option for these patients is to adopt a dietary style that includes foods with proven effects on the proper functioning of the immune system. In other words, one means of this adaptation is to establish a “pro-immunity menu” that fully coincides with the Mediterranean Dietary pattern [39].

### 4.2. Inflammatory State

#### 4.2.1. Continued Inflammatory Response

The inflammatory response is a normal physiological defense against pathogen infection and tissue damage. The basic physiological aspects that occur in the inflammatory process are firstly, the focalization of the response, which tends to circumscribe the area of fight against the aggressor agent. Secondly, the inflammatory response is of immediate action, of urgency and therefore, preponderantly non-specific, although it can favor the later development of a specific response. Thirdly, the inflammatory focus attracts immune cells from nearby tissues. The vascular alterations will also allow the arrival of immune mediators from the blood. Fourthly, when the causes of the aggression have disappeared or have been eliminated by the inflammatory response itself, repair processes are initiated and the inflammation ends rapidly [40]. However, in many chronic diseases, the inflammatory response continues and leads to significant tissue and organ damage. Thus, an abnormally prolonged and exacerbated inflammatory response would be closely related to the etiopathogenesis of some chronic diseases, especially of autoimmune origin, such as rheumatoid arthritis, inflammatory bowel disease, systemic lupus erythematosus, gout and diabetes [41].

SARS-CoV-2 infection triggers an inflammatory storm, the so-called “cytokine storm syndrome (CTS)”, by the whole virus or fragments of the virus, in its acute or quiescent phase. This event is an immunopathological feature of COVID-19 and has been associated with disease severity and with persistence of symptoms. CTS is a very severe, life-threatening complication of SARS-CoV-2 infection. After the initial early infection and pulmonary phase, some patients exhibit a hyperinflammatory response, with macrophage activation, mediated primarily through IL-1 and IL-6. Elevated C-reactive protein (CRP) and ferritin (FER) have been proposed as biomarkers to monitor the clinical course and determine treatment selection for patients with COVID-19 [42]. CTS generates high oxidative stress and thus involvement of the mitochondrial membrane, the organelle with which we are able to produce energy [41].

After this period of initial hyperinflammation induced by CTS, multisystem inflammatory syndrome (MIS) may appear 2–6 weeks after SARS-CoV-2 infection [26,28]. These patients present with elevated levels of systemic proinflammatory markers (CRP, IL-6, FER, and D-dimer) and/or vascular endothelium-related markers (associated with lung damage). In addition, these patients may present with severe shock, cardiac, gastrointestinal, or neurological symptoms and biomarkers. MIS suggests the involvement of a dysregulated adaptive immune system with a high degree of residual inflammation. Therefore, post-SARS-CoV-2 MIS symptoms may lead to LC [26]. Increased fluorine-18 Fluorodeoxyglucose (18F-FDG) uptake on positron emission tomography with multislice tomography (PET/CT) has been reported in patients who have overcome SARS-CoV-2 infection but with continued symptomatology for at least 1 month which could indicate persistent inflammation in the bone marrow and blood vessels [43]. Continued or unfinished inflammation could be one of the causes of the pathophysiology of LC, and could be associated with inflammation-related symptoms, myalgia, joint pain and fatigue [26]. 

SARS-CoV-2 induces pneumonia, characterized by the presence of lymphopenia and the existence of immune dysregulation and hyperinflation as an accompanying event of the critical illness caused by this virus [44]. The renewal and generation of B- and T-cell lymphocytes in an attempt to compensate for lymphopenia may generate elevated inflammation and contribute to LC [26]. In addition, lymphopenia correlates with persistent dissemination of SARS-CoV-2, which may further perpetuate chronic immune activation in LC [45].

#### 4.2.2. Gut Microbiota Imbalance

Pathological changes in the intestinal microbiome appear in numerous diseases of etiology associated with chronic inflammation processes. In addition, microbiota–gut–brain axis activity involves modulation of brain–gut neurotransmitter systems by the gut microbiome [46]. Gut dysbiosis has been observed among patients with COVID-19, lasting between 10–30 days after resolution of COVID-19. Intestinal dysbiosis was also correlated with increased severity of COVID-19 and increased plasma levels of inflammatory biomarkers and prolonged 6-week fecal excretion of SARS-CoV-2 [46,47]. That is, dysbiosis of the gut microbiota after disease resolution could contribute to the persistence of gastrointestinal and neurological symptoms in LC patients [48]. Therefore, it is necessary to study the role of the gastrointestinal tract and microbiota in the inflammatory processes of LC.

### 4.3. Viral Persistence

The infection produced by SARS-CoV-2 is systemic, affecting the entire body, rather than a single part or organ, as has been demonstrated in autopsies of patients where the presence of the virus has been found in multiple organs: lungs, pharynx, heart, liver, brain and kidneys. This establishes the possibility that there is a dissemination route, which could be through the peripheral nerves [49]. Systemic involvement and dissemination seem to contribute to viral persistence, causing latent or chronic infection. Viral persistence causes an immunological alteration to persist over time, resulting in chronic inflammation with long-lasting COVID-19 symptoms. In this sense, LC patients may not be able to completely eliminate SARS-CoV-2 after the acute infection phase due to an altered immune response [7]. The virus may remain latent in some reservoir (immune system tissue or cell) and periodically reactivate when changes in immune system homeostasis occur, causing outbreaks of symptomatology, but not making the virus detectable in the upper respiratory tract [49]. Therefore, persistence of SARS-CoV-2 in the body is possible, which may induce some level of immune activation contributing to an LC [8].

There is a history of viruses that do not insert into DNA and become chronic in certain subpopulations, such as hepatitis C virus, poliovirus, and Ebola virus [49]. With respect to SARS-CoV-2, cases have been reported of patients who remained positive for up to three months and even cases of prolonged dissemination in the respiratory tract for up to four months, evaluated by RT-PCR [26]. In addition, SARS-CoV-2 has been found to be hosting and distributed in various locations in the gastrointestinal tract, lungs, blood and in the olfactory mucosa where it would migrate to the central nervous system [49].

Recently, SARS-CoV-2 nucleic acids and proteins have been discovered in the small intestine of 50% of asymptomatic COVID-19 cases at 4 months after disease onset [22]. In addition, dissemination of SARS-CoV-2 in feces has also been detected for up to 8 weeks, even in the absence of gastrointestinal symptoms [50]. The persistence of SARS-CoV-2 at the intestinal level could be the origin of an unresolved inflammation process in the LC by active replication of SARS-CoV-2 in gastric and intestinal cells where there is an overexpression of ACE2 receptors. This situation could induce an increased fecal shedding of SARS-CoV-2 in patients [51]. In acute SARS-CoV-2 infection, gastrointestinal symptoms (loss of appetite, nausea, vomiting, diarrhea, and abdominal discomfort) affect between 10–20% of patients. In LC patients it affects more than 30%. Therefore, the persistence of SARS-CoV-2 in the gastrointestinal tract would be the cause of the gastrointestinal manifestations of LC [26].

#### Metabolic Alterations

Systemic or persistent SARS-CoV-2 infection exerts a significant impact on metabolism. Metabolomic analysis reveals abnormally elevated levels of ketone bodies (acetoacetic acid, 3-hydroxybutyric acid and acetone) and 2-hydroxybutyric acid (marker of hepatic oxidative stress), increases in hepatic glutathione synthesis and transaminase activity (aspartate aminotransferase (AST) and alanine aminotransferase (ALT)) and depletion of essential amino acids, tyrosine and glutamine. This corroborates that SARS-CoV-2 induces liver damage associated with dyslipidemia and oxidative stress [52]. There is a hypothesis that these alterations could also exist to some extent in LC, although it remains to be determined whether LC is a cause or a consequence of the disease and how it can be transferred to clinical practice, although it is surely an accompanying alteration and a reflection of the disease.

### 4.4. Coagulopathies

SARS-CoV-2-induced infection generates multi-organ dysfunction that triggers some coagulopathies that may result in hemorrhage and thrombocytopenia, hypercoagulation, pulmonary intravascular coagulation, microangiopathy, venous thromboembolism or arterial thrombosis. Acute COVID-19 infection is also characterized by dysregulated circulating inflammatory biomarkers such as the following: hyperactivated platelets, damaged erythrocytes, blood micro-clot accumulation in the lungs, increased D-dimer levels above twice normal, slightly prolonged prothrombin time (1–3 s above normal), and, late in the disease, decreased fibrinogen levels. Patients with acute COVID-19 may experience thrombocytopenia that can lead to life-threatening disseminated intravascular coagulation (DIC) [53,54].

The symptomatology of LC may be mainly due to the presence of persistent circulating blood clots from the acute phase that are resistant to fibrinolysis. Failure of the fibrinolytic process has been described during acute COVID-19 and in patients with LC symptoms. Plasma proteins in COVID-19 and LC plasma samples are highly resistant to degradation with trypsin. The formation of aberrant and fibrinolysis-resistant blood micro-clots caused by increased α2-antiplasmin (α2AP) or plasmin inhibitor antifibrinolytic action inhibiting the degradation of the fibrin networks of clots (for which plasmin is responsible). In addition, α2AP is associated with the mediation of inflammatory responses by stimulating the production of proinflammatory cytokines (IL-1, IL-6, TNF-α) that induce hypercoagulability and inflammatory immune responses. Dysregulation of mediators in the system in the circulation could be essential contributors in the pathophysiology of coagulation/multiple fibrinolysis of acute SARS-CoV-2 and LC infection [55]. Although there are significant differences of deregulated molecules when comparing samples from acute COVID-19 patients with patients with LC, which could be explained by the prolonged inflammatory state and persistent viral infection in LC [56]. Therefore, the development of LC could be associated with: (i) Development of hypercoagulability with significant increases in circulating inflammatory molecules; (ii) Presence of circulating micro-clotting and hyperactivation of thrombocytes; (iii) Generation of an aberrant fibrinolytic system, which induces hypo-fibrinolysis and persistent micro-clotting, in the presence of elevated levels of α2AP [56].

## 5. Clinical Characteristics of Long COVID

The clinical characterization should be made under the premise that LC is a multiorgan symptomatic complex that affects those patients who have suffered from COVID-19 by the persistence of symptoms beyond 4–12 weeks after infection with SARS-CoV-2, regardless of the severity of the acute phase and whether the diagnosis of the infection was made by laboratory or clinical tests (due to the low availability of tests), with a frequently fluctuating or flare-like presentation of symptoms, causing disability to the sufferer, without the existence of an explanation by an alternative underlying disease [57].

### 5.1. Incidence

Determining the incidence of LC is complex due to the novelty of the disease, the lack of rigorous clinical registries and the absence of specific surveillance of LC. In the community population (25–69 years) in the United Kingdom, it was estimated that 1 in 5 persons with COVID-19 had symptoms beyond 5 weeks (21% (95% CI 19.9–22.1)) and 1 in 10 beyond 12 weeks (9.9% (95% CI (6.7–14.79)). A higher incidence was reported in women (23.6% (95% CI 22.2–25.0)) than in men (20.7% (95% CI 19.3–22.1)) [58,59]. In a patient cohort of more than twenty thousand patients in the US population aged 18–89 years, 30% showed symptoms compatible with LC at 30 days, 25% at 60 days and 15% at 90 days after suffering a SARS-CoV-2 infection with diagnosis confirmed by RT-PCR [60]. Other studies [21,61] estimate the incidence of LC at 10% of those infected. When other populations are considered that include heterogeneous groups of patients (severe and very severe acute illness and hospitalized patients), prevalence’s of up to 80% of those affected appear [62]. It is necessary to consider that in these patients the true persistence of symptoms is mixed with the sequelae of the severe acute disease that produces structural damage and consequent clinical manifestations.

### 5.2. Symptomatology

**Figure 2:**
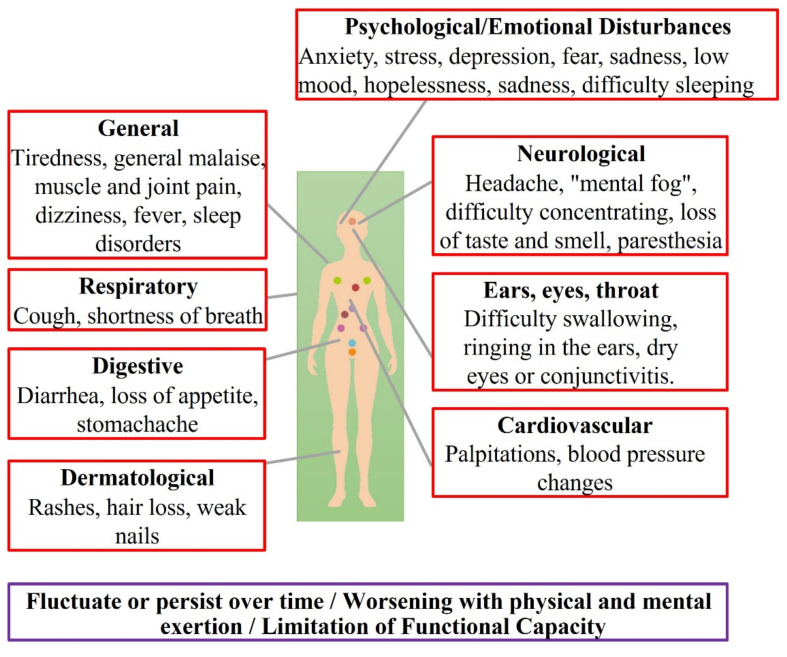
Symptomatology associated with Long COVID.

The symptoms and signs reported by people with LC are extremely numerous and varied (Figure 2) [63], which could perhaps be related to the different causes of the persistence of symptoms, which adds to the complexity of the syndrome in terms of its diagnosis, follow-up and its requirement for multidisciplinary health care [7]. Regarding the common symptomatology in LC patients, the following stand out: intermittent clinical presentation, exacerbation of symptoms with physical or mental effort and the so-called “mental fog”, which encompasses multiple symptoms associated with cognitive impairment such as memory loss, disorientation, interference in executive functions or problems in learning and concentration, and limitation of functional capacity [64].

The demographic profile of an LC patient is one of any age, sex, and condition. Half of the LC patients are between 36–50 years, 80% are women, and mostly without associated comorbidities prior to COVID-19. The mean persistence of symptoms is 3 to 6 months, the most frequent being fatigue, 77.7%, post-exertional malaise, 72.2%, and cognitive dysfunction, 55.4%; the majority of LC patients present general symptoms, 98.3%, neurological, 88.0%, and psychological/emotional, 88.3% [60,64,65].

A high percentage of patients with LC experience psychological or emotional disturbances such as the following: low mood, hopelessness, sadness, high levels of anxiety, difficulty sleeping, fear and stress. The etiology of the psychological consequences of SARS-CoV-2 infection could be multiple, stemming from the direct effects of viral infection, brain infection, cerebrovascular disease, medical interventions, social isolation, concern about infecting others, stigma, and the impact of a new disease. In addition, the source of emotional disturbances (distress, uncertainty, hopelessness, sadness, loneliness) may be part of a process of adaptation/acceptance to the new LC situation [60,64,65].

Skin lesions as a symptom in patients with LC are diverse and, in many cases, are reminiscent of skin lesions caused by other viruses such as Parvovirus B19_._ Papulosquamous eruptions and erythema pernio are the most common and could be triggered by persistent inflammatory states after acute SARS-CoV-2 infection. Other common dermatological lesions in LC are alopecia and “COVID toes”, and necrotic skin lesions related to the use of vasopressors or decubitus ulcers [66].

## 6. Pediatric Long COVID

The pediatric population is not the population group with the highest incidence or severity in the COVID-19 pandemic, although cases of symptomatology compatible with LC are beginning to be described in this age cohort [67]. In the pediatric population, the symptomatology of acute COVID-19 was mild in most patients. Symptoms of LC at 2–3 weeks after infection were: dermatological involvement [66], neurological, ophthalmological and psychiatric symptoms and to a lesser extent MIS [14]. A study in a Spanish population [68], adolescents or preadolescents, described LC with a “constitutional symptomatology” where persistent low-grade fever, intense asthenia and intense headache were its most frequent manifestations. Moreover, these symptoms were disabling for the children, as reported by their parents, and approximately 50% of the patients were seen in hospital emergency departments or primary care at least once but did not require hospital admission [68]. A study in Dutch patients aged 2 to 18 years [69], with suspected LC, reported symptomatology of fatigue (87%), dyspnea (55%) and concentration difficulties (45%). Of the sample of 89 patients, 36% of the children suffered severe limitations in the function of daily living [69]. In this regard, pediatric patients developed the main LC symptoms similar to those described in adults [70,71] (fatigue, headache, decreased physical capacity, cognitive impairment, decreased quality of life), and children were even unable to complete a school day 6 months after SARS-CoV-2 infection [70]. Moreover, persistence of LC symptoms can range from 3 to more than 5 months after resolution of COVID-19, showing 1 or 2 symptoms and 3 or more for 35.7% and 22.5% respectively of a sample of 129 Italian children [72]. Of note, in one of the first studies to report LC in children, based on a case series study, 4 out of 5 children were female [73].

## 7. What Is the Likelihood of Long COVID?

Vaccines against COVID-19 protect against this disease because they induce immunity against the SARS-CoV-2 virus that causes it, i.e., they reduce the risk of it causing symptoms and having health consequences. However, they do not prevent transmission of the virus [74]. Considering that approximately 1 in 5 people have symptoms 5 weeks after infection and 1 in 10 people have symptoms 12 weeks after infection [21,61], it is necessary to establish the probability of developing LC when SARS-CoV-2 infection has been confirmed to initiate the diagnostic approach, treatment, and follow-up of patients.

The development of more than five [75,76] or more than ten [77] symptoms during the first week of acute SARS-CoV-2 infection was associated with an increased risk of developing LC. These first-week symptoms were fatigue (97.7%), intermittent headache (91.2%), dyspnea, and anosmia [78]. Women with psychiatric pathology and/or treatment are risk factors for developing LC [79,80]. In addition, higher rates of LC have been reported in women than men after hospital discharge [26]. Therefore, the female sex may have a higher probability of developing LC. However, other authors describe that men and woman are equally likely to develop LC [71,77,81]. Other factors that could predict the occurrence of include old age (+70 years) and the presence of comorbidities [75,78]. In adults older than 70 years, loss of smell (which is less common) was the most predictive of LC (OR = 7.35), ahead of fever (OR = 5.51) and hoarse voice (OR = 4.03). Regarding the likelihood of developing LC, two studies [82,83,84] have reported no association between LC and the initial severity of acute COVID-19. However, being a critically ill patient or severe patient of acute SARS-CoV-2 infection, with long stay in the hospital or intensive care unit (ICU) with mechanical ventilation are risk factors [26,85]. 

## 8. Disability Associated with Long COVID

The disability of the LC process, together with that generated by its symptoms, translates into significant muscle loss and functional deficits that negatively impact quality of life. The most disabling symptoms are asthenia/fatigue, malaise, headache, muscle and joint pain, dyspnea, chest pressure/pain, decreased concentration and anosmia [64]. In Spain, the survey of symptoms and disability produced in LC patients [86] showed disability for daily tasks: household activities, personal hygiene, work activity, family obligations, and leisure activities. In patients affected by LC and who consider that it produces moderate or severe disability, it is necessary to analyze the impact on functionality, independence, and work capacity. The Baecke Physical Activity Short Questionnaire (BPAQ) test could be used to assess the average level of physical activity at work, sport, and leisure [87]. In addition, it is necessary to complete the assessment with quality-of-life scales, such as the SF-36 [88].

## 9. Basic Biomarkers in Long COVID

The evaluation of the LC patient should allow differential diagnosis with other pathologies presenting similar signs or symptoms. Currently, given that there is no specificity of laboratory tests in LC, biomarker evaluation should be based on physical examination findings and symptomatology [89]. Variations in D-dimer, CRP, and lymphocyte levels appeared consistent and may serve as possible biomarkers of LC [26,90]. However, other studies have found no changes in proinflammatory biomarkers between LC patients and patients without LC symptoms [80,81,82]. The differences may be due to the patient sample and methodology. In addition, it may be due to the wide variety of LC symptoms and their presentation in the form of recurrent flares [63]. This situation of multiple and fluctuating symptoms, in relation to disease activity and patient status, of inflammatory biomarkers is like those of autoimmune diseases and chronic inflammatory diseases [26,91].

Some scientific–medical societies and patient collectives have proposed a series of basic analytical parameters that should be evaluated after exceeding the 4–6 weeks in which acute SARS-CoV-2 infection should be resolved, but symptoms persist in patients [92]. The basic biomarkers to analyze would be the following: Complete blood count and erythrocyte sedimentation rate (ESR); Glucose; Lipid profile; Renal profile: creatinine, urea, glomerular filtrate; Ions: sodium, potassium; Liver profile: Bilirubin, ALT and AST, GGT, alkaline phosphatase; Albumin; LDH; CRP; Thyroid function tests; Iron metabolism; Vitamin B12, folate, Vitamin D; Minerals (calcium, phosphorus); Coagulation tests.

In addition, it should be completed with antibody determination tests and cellular immunity studies. Antibodies acting against SARS-CoV-2 can usually be detected in the first weeks of infection. The presence of antibodies indicates that the person was infected with SARS-CoV-2, regardless of the severity of the acute illness or even whether he or she was asymptomatic [93]. However, it has been observed that a certain percentage of patients with SARS-CoV-2 infection had no anti-SARS-CoV-2 IgG antibodies detected, with no certainty as to whether this is a lack of humoral immune response or a technical weakness. In approximately 10% of patients with mild cases of COVID-19, it is not possible to find anti-SARS-CoV-2 IgG. Moreover, this B-lymphocyte-mediated response is limited, and the antibodies may disappear over time. For this reason, the study of cell-mediated immunity is proposed [18,21]. The T cell response is of longer duration and allows the generation of a rapid response in case of a second contact with the virus. Recent studies have identified T cells reactive to SARS-CoV-2, both in severe cases of COVID-19 and in mild or asymptomatic cases [94]. The basic cellular immunity study may include the following immune biomarkers: IFN-γ, IL-2, B and T lymphocytes, CD14+ and CD16+, cellular response against SARS-CoV-2 antigens (S, M, N, P proteins), NK and CCL4 [92]. In addition, the FDA has recently validated the first test to detect specific cellular immunity: *T-Detect COVID test* developed by Adaptive Biotechnologies [95].

The Spanish Society of General and Family Physicians (SEMG) has implemented a basic care kit for the LC patient, which could help to collect the most relevant signs and symptoms in the study of LC [96]. This kit includes evaluation of symptoms and their evolution; Complete anamnesis and by systems/devices; Clinical evaluation scales; Physical examination; Complementary tests; Immunological and inflammatory biomarkers; Study of comorbidities; Physical health; Emotional health; Social health; Functional status [96].

## 10. Potential Treatment of Long COVID

No specific treatment for LC is currently available because of the great symptomatic variability, the multiple organs involved and knowledge of the pathophysiological mechanism causing the symptoms. The etiopathogenic origin of the symptoms could be considered for the therapeutic approach. Thus, viral persistence, exacerbated inflammatory state and immune hyperresponsiveness would be therapeutic targets in LC [7,26]. It is possible that several therapeutic targets may be used simultaneously, given the varied symptomatologic profile of the LC patient. The treatments described are hypotheses since the limited evidence available at the present time does not allow us to recommend their use. Potential pharmacological treatments would be aimed at treating clinical symptoms, i.e., experimental systemic treatments aimed at the etiological and integral treatment of the disease. This could include those that reestablish nutritional, metabolic, and intestinal flora balances, by means of drugs or nutritional supplements. In addition, local and/or symptomatic treatments could be used, based on current knowledge, for the most frequent and/or disabling symptoms. Two main groups of symptoms, physical symptomatology and emotional/cognitive symptomatology could be the best candidates for treatment. Symptomatic treatment can include changes to healthy lifestyle behaviors, pharmacological treatments targeting specific symptoms, physiotherapy, physical, olfactory and cognitive rehabilitation, psychological intervention, respiratory training, speech therapy, occupational therapy and prescription of physical exercise [92]. The practice of regular physical activity (PA) has been shown to be an effective therapy for most chronic diseases and microbial infections with preventive/therapeutic benefits, considering that exercise involves primary immune mediators and/or anti-inflammatory properties. These properties of PA would make it a useful complementary tool for prevention, which can also enhance recovery and improve the quality of life of patients affected by the different conditions resulting from COVID-19 infection [97]. Therefore, given the multisystemic nature of LC, multiple specialists should participate in the patient care process to implement therapeutic, rehabilitative, and individual care plans that facilitate comprehensive care of the patient with the aim of improving his or her quality of life.

## 11. Conclusions

The pandemic has brought us a wave of a new chronic and disabling disease called LC, which should be explored further. According to the data, so far, at least 10% of acute COVID-19 survivors develop LC. More specifically, 1 in 5 people have some symptoms after 5 weeks of infection and 1 in 10 people have some symptoms after 12 weeks of infection [21,61]. This leads to an estimate of approximately 6 million patients developing LC and its long-term consequences are worrisome because LC can affect regardless of the initial severity of the disease. This article could decrease uncertainty and aid the understanding of this new disease by addressing the background, involvement, mechanisms of action in the etiopathogenesis of the disease, the clinic, pediatric LC, the likelihood of developing LC, LC-associated disability, biomarkers for follow-up, and potential treatments. However, much remains unknown about LC, particularly its risk factors, its multiple symptomatic presentations, and pathophysiology, ranging from long-term damage to multiple organ systems, immune dysregulation, to unresolved inflammation of multiple origins. Therefore, LC remains a syndrome whose etiopathogenesis and treatment should be further investigated.

## Data Availability

Not applicable.

## References

[1] Lai C.C., Shih T.P., Ko W.C., Tang H.J., Hsueh P.R. (2020). Severe acute respiratory syndrome coronavirus 2 (SARS-CoV-2) and coronavirus disease-2019 (COVID-19): The epidemic and the challenges. Int. J. Antimicrob. Agents.

[2] Tabish S.A. (2020). COVID-19 pandemic: Emerging perspectives and future trends. J. Public Health Res..

[3] COVID-19 Map—Johns Hopkins Coronavirus Resource Center. https://coronavirus.jhu.edu/map.html.

[4] Fernández-Lázaro D., Fernandez-Lazaro C.I., Mielgo-Ayuso J., Adams D.P., García Hernández J.L., González-Bernal J., González-Gross M. (2021). Glycophosphopeptical AM3 Food Supplement: A Potential Adjuvant in the Treatment and Vaccination of SARS-CoV-2. Front. Immunol..

[5] Fernández-Lázaro D., Gómez N.S., Serrano N.S., Sosse A.A., Aldea-Mansilla C. (2020). Emergency Standardization for SARS-CoV-2 Virus Diagnosis by Real-Time Reverse Transcription-Reverse Polymerase Chain Reaction (RT-PCR) in a COVID-19 Pandemic Situation. Rev. Madrileña Salud Pública.

[6] Lai C.C., Ko W.C., Lee P.I., Jean S.S., Hsueh P.R. (2020). Extra-respiratory manifestations of COVID-19. Int. J. Antimicrob. Agents.

[7] Crook H., Raza S., Nowell J., Young M., Edison P. (2021). Long Covid-mechanisms, risk factors, and management. BMJ.

[8] Greenhalgh T., Knight M., A’Court C., Buxton M., Husain L. (2020). Management of post-acute Covid-19 in primary care. BMJ.

[9] World Health Organization (WHO) (2020). What We Know about Long-Term Effects of COVID-19. https://scholar.google.es/scholar?hl=es&as_sdt=0%2C5&q=What+we+know+about+long-term+effects+of+COVID-19.+The+latest+on+the+COVID-19+global+situation+and+long-term+sequelae&btnG=.

[10] Datta S.D., Talwar A., Lee J.T. (2020). A Proposed Framework and Timeline of the Spectrum of Disease Due to SARS-CoV-2 Infection: Illness Beyond Acute Infection and Public Health Implications. JAMA.

[11] The National Institute for Health and Care Excellence (NICE) (2021). COVID-19 Rapid Guideline: Managing the Long-Term Effects of COVID-19.

[12] Ministry of Health, Government of Spain, Health Alerts and Emergencies Coordination Center (2021). Contributions of This Update Scientific-Technical Information Coronavirus Disease, COVID-19. https://www.aemps.gob.es/https://www.mscbs.gob.es/profesionales/saludPublica/prevPromocion/vacunaciones/covid19/vacunasCovid19.htm.

[13] World Health Organization (WHO) (2021). What Is Health Policy and Systems Research. https://ahpsr.who.int/what-we-do/what-is-health-policy-and-systems-research-.

[14] Andrade B.S., Siqueira S., Soares W.R.d.A., Rangel F.d.S., Santos N.O., Freitas A.d.S., Silveira P.R.d., Tiwari S., Alzahrani K.J., Góes-Neto A. (2021). Long-COVID and Post-COVID Health Complications: An Up-to-Date Review on Clinical Conditions and Their Possible Molecular Mechanisms. Viruses.

[15] Proal A.D., VanElzakker M.B. (2021). Long COVID or Post-acute Sequelae of COVID-19 (PASC): An Overview of Biological Factors That May Contribute to Persistent Symptoms. Front. Microbiol..

[16] Rodríguez Ledo P., Armenteros del Olmo L., Guerrero Caballero S., Bilbao Fernández S. (2021). The persistence of COVID-19 symptoms and its diagnosis in the first wave of the pandemic in Spain. Med. Gen. Y Fam..

[17] Khan M., Adil S.F., Alkhathlan H.Z., Tahir M.N., Saif S., Khan M., Khan S.T. (2020). COVID-19: A Global Challenge with Old History, Epidemiology and Progress So Far. Molecules.

[18] World Health Organization (WHO) (2020). International Classification of Diseases, 11th Revision (IDC-11). https://icd.who.int/es.

[19] L´Assurance Maladie (ameli.fr) (2021). Symptômes Prolongés Suite à la Covid-19: Diagnostic et Prise en Charge. Médecin. https://www.ameli.fr/medecin/actualites/symptomes-prolonges-suite-la-covid-19-diagnostic-et-prise-en-charge.

[20] Chippa V., Aleem A., Anjum F. (2021). Post-Acute Coronavirus (COVID-19) Syndrome. https://www.ncbi.nlm.nih.gov/books/NBK570608/.

[21] Patterson B.K., Guevara-Coto J., Yogendra R., Francisco E.B., Long E., Pise A., Rodrigues H., Parikh P., Mora J., Mora-Rodríguez R.A. (2021). l Immune-Based Prediction of COVID-19 Severity and Chronicity Decoded Using Machine Learning. Front. Immunol..

[22] Gaebler C., Wang Z., Lorenzi J.C.C., Frauke Muecksch F., Finkin S., Tokuyama M., Cho A., Jankovic M., Schaefer-Babajew D., Oliveira D.Y. (2021). Evolution of antibody immunity to SARS-CoV-2. Nature.

[23] Ong S.W.X., Fong S.W., Young B.E., Chan Y.H., Lee B., Amrun S.N., Chee R.S.-L., Yeo N.K.-W., Tambyah P., Pada S. (2021). Persistent Symptoms and Association with Inflammatory Cytokine Signatures in Recovered Coronavirus Disease 2019 Patients. Open Forum Infect. Dis..

[24] Suárez Reyes A., Villegas Valverde C.A., Suárez Reyes A., Villegas Valverde C.A. (2020). Characteristics and specialization of the immune response in COVID-19. Rev. Fac. Med..

[25] Bergamaschi L., Mescia F., Turner L., Hanson A., Kotagiri P., Dunmore B.J., Ruffieux H., De Sa A., Oisin Huhn O., Wills M.R. (2021). Early Immune Pathology and Persistent Dysregulation Characterise Severe COVID-19. medRxiv.

[26] Yong S.J. (2021). Long COVID or post-COVID-19 syndrome: Putative pathophysiology, risk factors, and treatments. Infect. Dis..

[27] Zuo Y., Estes S.K., Ali R.A., Gandhi A.A., Yalavarthi S., Shi H., Sule G., Gockman K., Madison J.A., Zuo M. (2020). Prothrombotic autoantibodies in serum from patients hospitalized with COVID-19. Sci. Transl. Med..

[28] Wang E.Y., Mao T., Klein J., Dai Y., Huck J.D., Liu F., Zheng N.S., Zhou T., Israelow B., Wong P. (2021). Diverse Functional Autoantibodies in Patients with COVID-19. medRxiv Prepr. Serv. Heal Sci..

[29] Li Q., Wang B., Mu K., Zhang J.A. (2019). The pathogenesis of thyroid autoimmune diseases: New T lymphocytes—Cytokines circuits beyond the Th1-Th2 paradigm. J Cell Physiol..

[30] Lu D.T.W., Lee C.H., Chow W.S., Lee A.C.H., Tam A.R., Fong C.H.Y., Law C.Y., Hong Leung E.K., Wang To K.K., Beng Tan K.C. (2021). Thyroid Dysfunction in Relation to Immune Profile, Disease Status, and Outcome in 191 Patients with COVID-19. J. Clin. Endocrinol. Metab..

[31] Dalli J. (2017). Does promoting resolution instead of inhibiting inflammation represent the new paradigm in treating infections?. Mol. Asp. Med..

[32] Serhan C.N. (2017). Treating inflammation and infection in the 21st century: New hints from decoding resolution mediators and mechanisms. FASEB J..

[33] Regidor P.A., Santos F.G., Rizo J.M., Egea F.M. (2020). Pro resolving inflammatory effects of the lipid mediators of omega 3 fatty acids and its implication in SARS COVID-19. Med. Hypotheses.

[34] Regidor P.A. (2020). Covid-19 management with inflammation resolving mediators? Perspectives and potential. Med. Hypotheses.

[35] Kumar R., Rathi H., Haq A., Wimalawansa S.J., Sharma A. (2021). Putative roles of vitamin D in modulating immune response and immunopathology associated with COVID-19. Virus Res..

[36] Rogero M.M., de Leão M.C., Santana T.M., de Pimentel M.V.M.B., Carlini G.C.G., Silveira T.F.F., Gonçalves R.C., Castro I.A. (2020). Potential benefits and risks of omega-3 fatty acids supplementation to patients with COVID-19. Free Radic. Biol. Med..

[37] Clemente-Suárez V.J., Ramos-Campo D.J., Mielgo-Ayuso J., Dalamitros A.A., Nikolaidis P.A., Hormeño-Holgado A., Tornero-Aguilera J.F. (2021). Nutrition in the Actual COVID-19 Pandemic. A Narrative Review. Nutrients.

[38] Shakoor H., Feehan J., Dhaheri A.S., Al Ali H.I., Platat C., Ismail L.C., Apostolopoulos V., Stojanovska L. (2021). Immune-boosting role of vitamins D, C, E, zinc, selenium and omega-3 fatty acids: Could they help against COVID-19?. Maturitas.

[39] Maraver-Romero R. (2020). Could the Mediterranean lifestyle prevent complications from Covid-19 infection?. Actual. Med..

[40] Haig D.M.K., McInnes C., Deane D., Reid H., Mercer A. (1997). The immune and inflammatory response to orf virus. Comp. Immunol. Microbiol. Infect. Dis..

[41] Duan L., Rao X., Sigdel K.R. (2019). Regulation of inflammation in autoimmune disease. J. Immunol. Res..

[42] Mehta P., McAuley D.F., Brown M., Sanchez E., Tattersall R.S., Manson J.J. (2020). COVID-19: Consider cytokine storm syndromes and immunosuppression. Lancet.

[43] Sollini M., Ciccarelli M., Cecconi M., Aghemo A., Morelli P., Gelardi F., Chiti A. (2021). Vasculitis changes in COVID-19 survivors with persistent symptoms: An [18F]FDG-PET/CT study. Eur. J. Nucl. Med. Mol. Imaging.

[44] Siddiqi H.K., Mehra M.R. (2020). COVID-19 illness in native and immunosuppressed states: A clinical–therapeutic staging proposal. J. Heart Lung Transpl..

[45] Liu B., Han J., Cheng X., Yu L., Zhang L., Wang W., Ni L., Wei C., Yafei Huang Y., Cheng Z. (2020). Reduced numbers of T cells and B cells correlates with persistent SARS-CoV-2 presence in non-severe COVID-19 patients. Sci. Rep..

[46] Belkaid Y., Hand T.W. (2014). Role of the microbiota in immunity and inflammation. Cells.

[47] Zuo T., Zhan H., Zhan F., Liu Q., Tso E.Y., Lui G.C., Chen N., Li A., Lu W., Chan F.K.L. (2020). Alterations in Fecal Fungal Microbiome of Patients with COVID-19 during Time of Hospitalization until Discharge. Gastroenterology.

[48] Marazzato M., Ceccarelli G., d’Ettorre G. (2021). Dysbiosis in SARS-CoV-2–Infected Patients. Gastroenterology.

[49] Guerrero Caballero S., Bilbao Fernández S. (2021). Persistence of SARS-CoV-2 virus as an etiologic cause of long-lasting symptomatology in patients with persistent COVID-19. Med. Gen. Y Fam..

[50] Wu Y., Guo C., Tang L., Hong Z., Zhou J., Dong X., Yin H., Xiao Q., Tang Y., Qu X. (2020). Prolonged presence of SARS-CoV-2 viral RNA in faecal samples. Lancet Gastroenterol. Hepatol..

[51] Xiao F., Tang M., Zheng X., Liu Y., Li X., Shan H. (2020). Evidence for Gastrointestinal Infection of SARS-CoV-2. Gastroenterology.

[52] Bruzzone C., Bizkarguenaga M., Gil-Redondo R., Diercks T., Arana E., García de Vicuña A., Seco M., Bosch A., Palazón A., San Juan I. (2020). SARS-CoV-2 Infection Dysregulates the Metabolomic and Lipidomic Profiles of Serum. iScience.

[53] Gupta A., Madhavan M.V., Sehgal K., Nair N., Mahajan S., Sehrawat T.S., Bikdeli B., Ahluwalia N., Ausiello J.C., Wan E.Y. (2020). Extrapulmonary manifestations of COVID-19. Nat. Med..

[54] Lee S.G., Fralick M., Sholzberg M. (2020). Coagulopathy associated with COVID-19. CMAJ.

[55] Becker R.C. (2020). COVID-19 update: Covid-19-associated coagulopathy. J. Thromb. Thrombolysis.

[56] Pretorius E., Vlok M., Venter C., Bezuidenhout J.A., Laubscher G.J., Steenkamp J., Kell D.B. (2021). Persistent clotting protein pathology in Long COVID/Post-Acute Sequelae of COVID-19 (PASC) is accompanied by increased levels of antiplasmin. Cardiovasc. Diabetol..

[57] Higgins V., Sohaei D., Diamandis E.P., Prassas I. (2021). COVID-19: From an acute to chronic disease? Potential long-term health consequences. Crit. Rev. Clin. Lab. Sci..

[58] Office for National Statistics (2020). The Prevalence of Long COVID Symptoms and COVID-19 Complications. https://www.ons.gov.uk/news/statementsandletters/theprevalenceoflongcovidsymptomsandcovid19complications.

[59] Office for National Statistics (2021). Updated Estimates of the Prevalence of Prolonged COVID-19 Symptoms. https://www.ons.gov.uk/peoplepopulationandcommunity/healthandsocialcare/healthandlifeexpectancies/adhocs/12788updatedestimatesoftheprevalenceoflongcovidsymptoms.

[60] Cirulli E.T., Barrett K.M.S., Riffle S., Bolze A., Neveux I., Dabe S., Grzymski J.J., Lu J.T., Washington N.L. (2020). Long-term COVID-19 symptoms in a large unselected population. medRxiv.

[61] Rajan S., Khunti K., Alwan N., Steves C., MacDermott N., Morsella A., Angulo E., Winkelmann J., Bryndová L., Fronteira  I. (2021). The Wake of the Pandemic: Preparing for Long COVID.

[62] Lopez-Leon S., Wegman-Ostrosky T., Perelman C., Sepulveda R., Rebolledo P.A., Cuapio A., Villapol S. (2021). More than 50 long-term effects of COVID-19: A systematic review and meta-analysis. Sci. Rep..

[63] Ministry of Health Government of Spain (2021). Clinical Information COVID-19. https://www.mscbs.gob.es/en/profesionales/saludPublica/.

[64] Davis H.E., Assaf G.S., McCorkell L., Wei H., Low R.J., Re’em Y., Signe Redfield S., Austin J.-P., Akrami A. (2021). Characterizing long COVID in an international cohort: 7 months of symptoms and their impact. EClinicalMedicine.

[65] Rodríguez Ledo P., Armenteros del Olmo L., Rodríguez Rodríguez E., Gómez Acebo F. (2021). Description of the 201 symptoms of multiorgan involvement in patients affected by persistent COVID-19. Med. Gen. Y Fam..

[66] Fernández-Lázaro D., Garrosa M. (2021). Identification, Mechanism, and Treatment of Skin Lesions in COVID-19: A Review. Viruses.

[67] Martins M.M., Prata-Barbosa A., Ledo Alves da Cunha A.J. (2021). Update on SARS-CoV-2 infection in children. Paediatr. Int. Child Health.

[68] Nogueira López J., Grasa C., Calvo C., García López-Hortelano M. (2021). Long-term symptoms of COVID-19 in children. Acta Paediatr..

[69] Brackel C.L., Lap C.R., Buddingh E.P., van Houten M.A., van der Sande L.J., Langereis E.J., Bannier M.A.G.E., Pijnenburg M.W.H., Hashimoto S., Suzanne W. J. (2021). Pediatric long-COVID: An overlooked phenomenon?. Pediatr. Pulmonol..

[70] Ludvigsson J.F. (2021). Case report and systematic review suggest that children may experience similar long-term effects to adults after clinical COVID-19. Acta Paediatr..

[71] Petersen M.S., Kristiansen M.F., Hanusson K.D., Danielsen M.E., Gaini S., Strøm M., Marin Strøm M., Weihe P. (2020). Long COVID in the Faroe Islands—A longitudinal study among non-hospitalized patients. Clin. Infect. Dis..

[72] Buonsenso D., Munblit D., De Rose C., Sinatti D., Ricchiuto A., Carfi A., Valentini P. (2021). Preliminary evidence on long COVID in children. Acta Paediatr..

[73] Radtke T., Ulyte A., Puhan M.A., Kriemler S. (2021). Long-term symptoms after SARS-CoV-2 infection in children and adolescents. JAMA.

[74] Sadarangani M., Marchant A., Kollmann T.R. (2021). Immunological mechanisms of vaccine-induced protection against COVID-19 in humans. Nat. Rev. Immunol..

[75] Sudre C.H., Murray B., Varsavsky T., Graham M.S., Penfold R.S., Bowyer R.C., Capdevila Pujol J., Klaser K., Antonelli M., LCanas L.S. (2021). Attributes and predictors of long COVID. Nat. Med..

[76] Maxwell E. (2021). Unpacking post-covid symptoms. BMJ.

[77] Stavem K., Ghanima W., Olsen M.K., Gilboe H.M., Einvik G. (2021). Persistent symptoms 1.5–6 months after COVID-19 in non-hospitalised subjects: A population-based cohort study. Thorax.

[78] Fernández-Lázaro D., Seco-Calvo J., Fernandez-Lazaro C.I., Sánchez-Serrano N. (2021). XVII Scientific Meeting: Science, technology and innovation at the service of the pandemic response plan in Colombia. The puzzling new condition associated with SARS-CoV-2 infection: Long COVID. Oral Commun. Biomed..

[79] Poyraz B.Ç., Poyraz C.A., Olgun Y., Gürel Ö., Alkan S., Özdemir Y.E., Balkan I.I., Karaali R. (2021). Psychiatric morbidity and protracted symptoms after COVID-19. Psychiatry Res..

[80] Townsend L., Dyer A.H., Jones K., Dunne J., Mooney A., Gaffney F., O’Connor L., Leavy D., O’Brien K., Dowds J. (2020). Persistent fatigue following SARS-CoV-2 infection is common and independent of severity of initial infection. PLoS ONE.

[81] Moreno-Pérez O., Merino E., Leon-Ramirez J.M., Andres M., Ramos J.M., Arenas-Jiménez J., Asensio S., Sanchez R., Ruiz-Torregrosa P., Galan I. (2021). Post-acute COVID-19 syndrome. Incidence and risk factors: A Mediterranean cohort study. J. Infect..

[82] Miyazato Y., Morioka S., Tsuzuki S., Akashi M., Osanai Y., Tanaka K., Terada M., Suzuki M., Kutsuna S., Saito S. (2020). Prolonged and Late-Onset Symptoms of Coronavirus Disease 2019. Open Forum Infect. Dis..

[83] Yelin D., Margalit I., Yahav D., Runold M., Bruchfeld J. (2021). Long COVID-19—It’s not over until?. Clini. Microbiol. Infect..

[84] Van den Borst B., Peters J.B., Brink M., Schoon Y., Bleeker-Rovers C.P., Schers H., van Hees H.W.H., van Helvoort H., van den Boogaard M., van der Hoeven H. (2020). Comprehensive health assessment three months after recovery from acute COVID-19. Clin. Infect. Dis..

[85] Taboada M., Cariñena A., Moreno E., Rodríguez N., Domínguez M.J., Casal A., Vanessa Riveiro V., Diaz-Vieito M., Valdés L., Álvarez J. (2021). Post-COVID-19 functional status six-months after hospitalization. J. Infect..

[86] Spanish Society of General and Family Physicians (SEMG) (2021). Survey of Symptoms and Disability Produced by the Same, in Those Affected by Persistent COVID. https://www.semg.es.

[87] Baecke J.A., Burema J., Frijters J.E. (1982). A short questionnaire for the measurement of habitual physical activity in epidemiological studies. Am. J. Clin. Nutr..

[88] Vilagut G., Ferrer M., Rajmil L., Rebollo P., Permanyer-Miralda G., Quintana J.M., Rosalía Santeda R., Valderasa J.M., Riberad A., Domingo-Salvanya A. (2005). The Spanish SF-36 Health Questionnaire: A decade of experience and new developments by researchers of the Red-IRYSS. Gac. Sanit..

[89] Doykov I., Hällqvist J., Gilmour K.C., Grandjean L., Mills K., Heywood W.E. (2020). “The long tail of Covid-19”—The detection of a prolonged inflammatory response after a SARS-CoV-2 infection in asymptomatic and mildly affected patients. F1000Research.

[90] Cojocarum M., Cojocarum I.M., Silos I., Vrabie C.D. (2011). Manifestations of Systemic Lupus Erythematosus. Mædica.

[91] Li H., Liu S.-M., Yu X.-H., Tang S.-L., Tang C.-K. (2020). Coronavirus disease 2019 (COVID-19): Current status and future perspectives. Int. J. Antimicrob. Agents.

[92] Spanish Society of General and Family Physicians (SEMG) (2021). Clinical Guideline for the Care of the Persistent Long COVID/COVID Patient. https://www.semg.es/index.php/consensos-guias-y-protocolos/363-guia-clinica-para-la-atencion-al-paciente-long-covid-covid-persistente.

[93] Muench P., Jochum S., Wenderoth V., Ofenloch-Haehnle B., Hombach M., Strobl M., Henrik Sadlowski H., Sachse C., Torriani G., Isabella Eckerle I. (2020). Development and Validation of the Elecsys Anti-SARS-CoV-2 Immunoassay as a Highly Specific Tool for Determining Past Exposure to SARS-CoV-2. J. Clin. Microbiol..

[94] Braun J., Loyal L., Frentsch M., Wendisch D., Georg P., Kurth F., Hippenstiel S., Dingeldey M., Kruse B., Fauchere F. (2020). SARS-CoV-2-reactive T cells in healthy donors and patients with COVID-19. Nature.

[95] US Food and Drug (FDA) (2021). Coronavirus (COVID-19) Update: FDA Authorizes Adaptive Biotechnologies T-Detect COVID Test. FDA. https://www.fda.gov/news-events/press-announcements/coronavirus-covid-19-update-fda-authorizes-adaptive-biotechnologies-t-detect-covid-test.

[96] Spanish Society of General and Family Physicians (SEMG) (2021). Basic Care Kit for Patients with Persistent COVID-19 Disease. https://semst.org/kit-de-atencion-basica-al-paciente-con-enfermedad-covid-19-persistente/.

[97] Fernández-Lázaro D., González-Bernal J.J., Sánchez-Serrano N., Navascués L.J., Ascaso-del-Río A., Mielgo-Ayuso J. (2020). Physical Exercise as a Multimodal Tool for COVID-19: Could It Be Used as a Preventive Strategy?. Int. J. Environ. Res. Public Health.

